# What Is Disease?

**Published:** 1888-11

**Authors:** J. H. Hanaford


### WHAT IS DISEASE?

In a general sense, disease is the legitimate and necessary result of the violations of the laws of our physical being—the condition of health. It is reasonable to infer that there is no pain, sickness and but few deaths which do not result from infringements of these laws—health being in a certain sense natural, and disease accidental, inflicted in the line of penalty.

We may conclude that the great and good Father is best pleased with His children when they regard all of His laws, including, most certainly, the laws of the body, established for the regulation of that body, by a proper observance of which a natural condition of good health can be secured—our health being as certainly under our control, and to as great an extent as any branch of our business or employment, or our education —while disobedience, and consequent physical suffering and disease, not only are not in accordance with His pleasure and design, but such violations of His laws will constitute sin, as certainly as a violation of a moral law, both having the same divine origin.

The true condition of the advanced man is that in which his whole being is harmoniously developed, the body so cared for and fed that it will have some of the vigor and eudurance of its original state, as he came from the hand of the Creator, when, as one of the best thinkers of the age in which he lived, Horace Mann, said of man before he fell, physically, by gross violations of organic laws, "He was so perfect in his bodily organs, so defiant of cold and heat, of drought and humidity, so sur-charged with vital force, that it took more than two thousand years of the combined abominations of appetite and ignorance ; it took successive ages of outrageous excess and debauchery to drain off his electric energies and make him even accessible to disease ; then it took ages more to breed all of these vile distempers which now nestle, like vermin, in every fiber of the body ! " Although suffering, tortures and disease follow in the direct line of penalty for disobedience, a disregard of necessary laws of our being, these penalties are administered in mercy, what we call diseases being, generally, only efforts of nature to avert the worst results of our wrong doings, and to improve the general condition of the system.

We may have a cough, yet that is not the real difficulty, but the result of a struggle of the recuperative powers, the vital energies, to dispose of certain accumulations which would otherwise prove harmful, if not fatal. The duty of the nurse, physician, etc., is to co-operate with nature in this friendly effort for improvement and purification, "loosen the cough" and promoting expectoration. Yet some young practitioners, who suppose that they are saturated with science, do the best (or worse) that they can to antagonize nature, crippling her in every respect by the administration of opiates, retaining such foul accumulations to serve as irritants in future. Yet, it is well known that, in the advanced stage of lung affections, when expectoration is impossible, when the system is measurably contaminated, death soon follows.

Again, when more food is eaten than can be digested, the remainder ferments, decays, putrifies in the stomach, threatening harm to the whole system. A nausea is instituted, followed by convulsive efforts of nature to expel the putrid and poisonous accumulation in mercy, which we call disease—vomiting—which should always be encouraged, "rinsing out" the stomach with warm water till cleanliness is secured. Yet, opiates are often given to foil nature in her merciful efforts for purification, and these efforts of the young practitioner to antagonize the recuperative powers too often proving a partial success. The resultant disease, so-called, may be a flux; nature, foiled in her attempt to rid the stomach of its poisonous burden, by vomiting, *hustles* the mass into the bowels, where another effort at expulsion is instituted. Nature is generally prompt in such measures, doing the best that can be done, under the circumstances, at least, unaided. By the use of opiates this merciful effort of nature may be suspended, the impurities retained in the body, yet nature is not so easily pacified, not easily conquered. The next effort to purify may be to produce cutaneous diseases, throwing the visceral impurities to the surface, as the next most available means of avoiding worse diseases. Just to the extent that these discharges are effected, health may be secured, the system purified. If astringents are applied, the discharges checked, in consequence of the foolish fear of "running the life away," when nothing but impurities, poisons, are discharged—the more the better—outraged nature may still attempt a cure, a purification, by instituting a fever, which the meddlesome young practitioner cannot so easily control. If the causes are not removed the fever will be quite sure to have its own course, taking its time for the renovation and cure. The vital energies quicken the circulation, sending the blood to the lungs for purification, these two acts energizing the whole system. What nature failed to expel, in consequence of the astringents, as the next resort is actually burned by feverish action, the combustion producing the heat of the fever. Such a fever, if not troubled by intruders, consumes vast quantities of effete matter, purifying and restoring health.

If our Heavenly Father punishes us for our physical sins, our reckless disregard of the physical laws which he instituted for the protection of our health and the promotion of our physical welfare He supplemented such penalties with reconstructive instrumentalities, recuperative measures, conducive to our real good. If we suffer, it is because we are wrong; we outrage our physical being. It would be blasphemous to charge the good Father with creating idiots, monsters, the physically dwarfed and diseased, surcharged with the overflowing *rottenness* of licentiousness, the gangrenous and the deformed, the blind and the halt, the thousands of the victims of degraded and vicious parents, in whom the foul emanations, in the form of malignant diseases, are constantly outcropping from week to week, rendering mortal life but a fearful series of plagues and epidemics, the diseased bodies scarcely being capable of containing the constant stream of foulness which naturally flows from a vicious and licentious life down to a sin-cursed progeny. No, if we suffer it is because we produce sufferings, create our diseases ! !—Dr. J. H. Hanaford, in *The Phrenological Journal.*

